# Association Between Temozolomide Resistance and Long Non-Coding RNA Expression Profiles in Glioblastoma Cell Lines

**DOI:** 10.3390/ph19081159

**Published:** 2026-07-25

**Authors:** Zuzana Hudáková, Ľuboš Hudák, Zuzana Hatoková, Peter Račay, Jozef Hatok

**Affiliations:** 1 Department of Medical Biochemistry, Jessenius Faculty of Medicine in Martin, Comenius University in Bratislava, Mala Hora 11161/4D, SK-03601 Martin, Slovakia; novakiova2@uniba.sk (Z.H.);; 2 Biomedical Centre Martin, Jessenius Faculty of Medicine in Martin, Comenius University in Bratislava, Mala Hora 11161/4C, SK-03601 Martin, Slovakia

**Keywords:** glioblastoma, temozolomide, resistance model, lncRNA

## Abstract

**Background:** Glioblastoma (GBM) is the most aggressive primary brain tumour in adults and is characterised by poor prognosis and frequent resistance to temozolomide (TMZ)-based chemotherapy. Increasing evidence suggests that long non-coding RNAs (lncRNAs) contribute to GBM progression, therapeutic adaptation, and chemoresistance. This study investigated the relationship between TMZ responsiveness and the expression of selected lncRNAs in GBM cell lines exhibiting distinct sensitivity profiles. **Methods:** Human GBM cell lines (A172, U87, and T98G) and normal human astrocytes (NHA) were exposed to TMZ for 24–72 h. Cell viability, cell cycle distribution, MGMT protein expression, and expression levels of nine selected lncRNAs were analysed using MTT assay, flow cytometry, Western blot, and quantitative RT-PCR, respectively. **Results:** TMZ induced dose- and time-dependent reductions in cell viability in all analysed cell lines. A172 cells exhibited the greatest TMZ sensitivity, whereas T98G cells displayed the highest resistance. TMZ-sensitive GBM cells demonstrated pronounced G2/M cell cycle arrest, while T98G cells showed minimal cell cycle perturbation. MGMT protein expression was markedly elevated in T98G cells and decreased following exposure to higher TMZ concentrations. Distinct lncRNA expression profiles were identified among GBM cell lines. MALAT1 expression was consistently reduced, whereas NCK1-AS1 was strongly upregulated, particularly in T98G cells. TMZ exposure induced significant alterations in H19, PVT1, OIP5-AS1, and NCK1-AS1 expression, suggesting their potential involvement in adaptive resistance mechanisms. **Conclusions:** Collectively, these findings indicate that selected lncRNAs, particularly H19, MALAT1, NCK1-AS1, and PVT1, may contribute to TMZ resistance in GBM and could be promising biomarkers and therapeutic targets for precision oncology approaches.

## 1. Introduction

Gliomas, which are tumours that originate from neuroglial progenitor or stem cells, are the most prevalent primary tumours of the brain and spinal cord in adults, accounting for more than 80% of all intracranial tumours [1,2]. Diverse glioma types exist, which is why efforts to establish a unified classification system have been ongoing since 2007. The current fifth edition of the World Health Organization Classification of Tumors of the Central Nervous System (WHO CNS5), published in 2021, categorises gliomas on the basis of not only their histological features but also their molecular characteristics, genetic aetiology, and prognostic factors [3,4]. This classification indicates that the majority of the most aggressive primary brain tumours of adults fall into the family of adult-type diffuse gliomas, with glioblastoma (GBM), IDH-wildtype, being the most aggressive [3,5,6]. GBM is considered the deadliest form of glioma, with a five-year survival ranging from 0.05% to 4.7% [1] and a median overall survival of less than six months after diagnosis. This period can be extended to 12–15 months with standard therapy following the Stupp regimen, which consists of surgical resection of the maximal safe margin of the tumour, followed by adjuvant radiotherapy and chemotherapy [7,8]. Currently, the cornerstone of GBM therapy is the chemotherapeutic prodrug temozolomide (TMZ) [9,10].

The antitumor mechanism of TMZ lies in the methylation of purine bases, resulting in the formation of N7-methylguanine (70%), N3-methyladenine (9%), and the cytotoxic O6-methylguanine (6%) [11]. Methylation at the O6 position of guanine is the primary cytotoxic event, which results in mismatched base pairing during DNA replication, thus causing double-stranded breaks, arrest of the cell cycle in the G2/M phase, and, ultimately, cell death [12,13,14]. Even though TMZ has been introduced as a first-line therapeutic for GBM, its limited efficacy and the development of resistance in the majority (approximately 90%) of recurrent GBM are persistent challenges [11,12,15]. One of the main prognostic factors that affect TMZ resistance is the methylation status of the O6-methylguanine-DNA methyltransferase (MGMT) promoter. The DNA repair enzyme MGMT reverses the cytotoxic O6-methylguanine induced by TMZ; hence, hyper-methylation of the MGMT promoter is associated with improved patient survival [11,16]. Various recent studies have focused on the TMZ resistance of GBM cells by comparing established TMZ-resistant cell lines with well-known TMZ-sensitive cells, often centring on MGMT promoter methylation or genomic alterations in p53 [15,16,17,18].

Similarly to other tumours, GBM is considered a genetic disease, with two main categories of genes involved in its development: proto-oncogenes and anti-oncogenes, also known as tumour suppressor genes [19,20]. Proteins such as epidermal growth factor receptor, platelet-derived growth factor, p53, and phosphatase and tensin homolog have been identified as crucial players in glioma development [19]. In recent years, numerous research groups have highlighted RNAs as versatile regulators of majority of cellular processes, further deviating from past assumptions that their role lies primarily in mediating the translation of DNA into protein [21]. Advances in RNA sequencing and mapping of transcriptomes revealed that only a minor fraction of RNA (approximately 2%) has protein-coding potential. The remainder of the transcriptome consists of non-coding RNAs, with those longer than 200 nt referred to as long non-coding RNAs (lncRNAs) [21,22,23,24]. Interest in the role of lncRNAs in gliomas and other tumours has increased in recent years, with multiple studies focusing on the impact of these molecules on tumour progression and treatment resistance [23,25,26]. Some studies propose a classification of lncRNAs in glioma similar to that of proteins, mostly dividing lncRNAs into two groups: oncogenic and tumour-suppressor lncRNAs. However, the distinction between these groups is often blurred because of the heterogeneity of cells that compose GBM [27]. Presently, lncRNAs are recognised as key regulators of multiple processes in GBM, including proliferation, invasion, angiogenesis, stemness, modulation of immune response, radiation therapy, and chemotherapy resistance [2,19,22,23,26,27,28]. Collectively, these findings indicate that lncRNAs play multifaceted roles in mediating TMZ resistance through epigenetic, transcriptional, and post-transcriptional mechanisms, making them promising therapeutic targets in GBM.

This study aimed to investigate the relationship between TMZ responsiveness and the expression of selected lncRNAs in GBM cell lines with distinct sensitivity profiles. Candidate lncRNAs were selected based on a comprehensive literature review combined with publicly available transcriptomic studies of glioblastoma. Selection criteria included: (i) reported dysregulation in glioblastoma tissues or cell lines, (ii) previously described involvement in TMZ response or resistance, (iii) participation in pathways related to proliferation, apoptosis, DNA damage response, stemness, or invasion, and (iv) reproducible differential expression reported in multiple independent studies, including analyses derived from TCGA, CGGA and GEO datasets. By integrating analyses of cell viability, cell cycle progression, MGMT expression, and lncRNA regulation, this work provides insight into potential molecular mechanisms associated with TMZ resistance in GBM. Our results demonstrate that four (H19, MALAT1, NCK1-AS1, and PVT1) of the nine analysed lncRNAs are associated with TMZ exposure, with notable cell type-specific effects.

## 2. Results

### 2.1. Cell Viability

**Figure 1:**
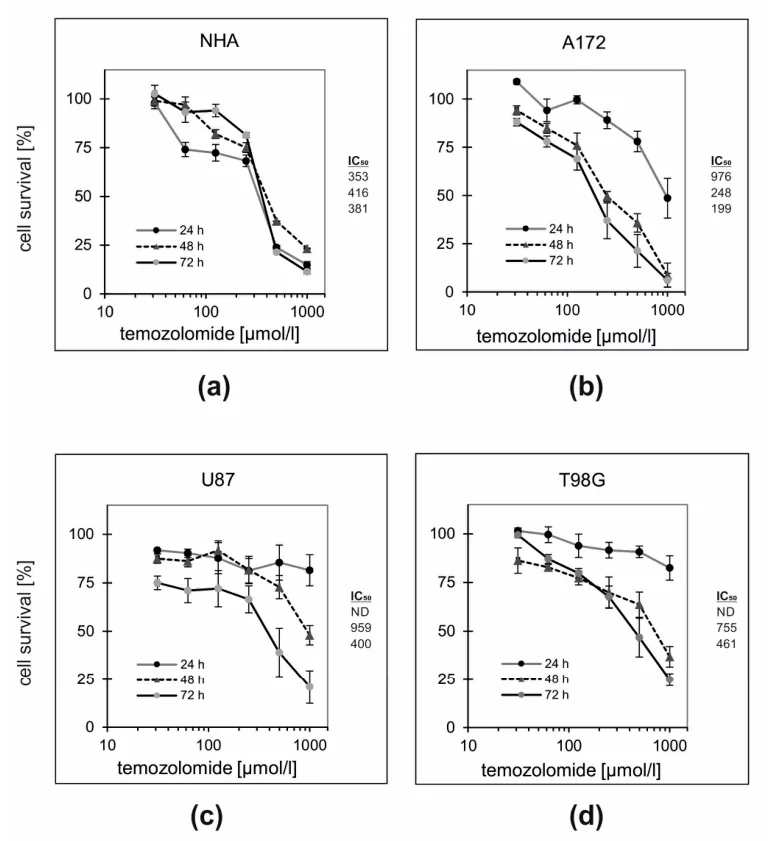
Dose response curves of cell survival in cell lines following treatment with TMZ. Data show a time- and dose-dependent decrease in cell viability, with corresponding IC_50_ values indicated for each condition; ND—not determined. Data are presented as mean ± SEM of three independent experiments.

The cytotoxic effect of TMZ on cell viability was evaluated in one normal human astrocyte (NHA) cell line and three GBM cell lines (A172, U87, and T98G) following 24, 48, and 72 h (h) of exposure. In the NHA cell line, TMZ induced a dose-dependent decrease in cell survival across all time points. However, the effect remained moderate at lower concentrations, with a more pronounced reduction observed only at higher doses. The IC_50_ values were relatively consistent over time (Figure 1a), indicating limited time-dependent sensitisation. In the A172 cells, TMZ exhibited a stronger cytotoxic effect than NHA did, particularly with prolonged exposure. Cell survival decreased progressively with both increasing concentration and time. Notably, the IC_50_ decreased substantially from 976 µmol/L at 24 h to 199 µmol/L at 72 h (Figure 1b), suggesting increased sensitivity over time. By contrast, the IC_50_ values for the known TMZ-resistant U87 and T98G cell lines at 24 h could not be determined (Figure 1c,d). Nevertheless, prolonged exposure of T98G cells to TMZ led to increased sensitivity, with IC_50_ values of 755 µmol/L at 48 h and 461 µmol/L at 72 h (Figure 1d). Despite this, T98G remained more resistant compared with A172 and U87 cells.

A172 cells exhibited the greatest sensitivity to TMZ, with progressive reductions in viability following prolonged exposure. By contrast, T98G cells displayed a markedly resistant phenotype, characterised by sustained survival and higher IC_50_ values throughout the treatment period. U87 cells demonstrated an intermediate response profile, whereas normal human astrocytes exhibited comparatively lower sensitivity. Overall, these findings demonstrate that TMZ responsiveness among GBM cell lines exhibits substantial heterogeneity.

### 2.2. Cell Cycle

**Figure 2:**
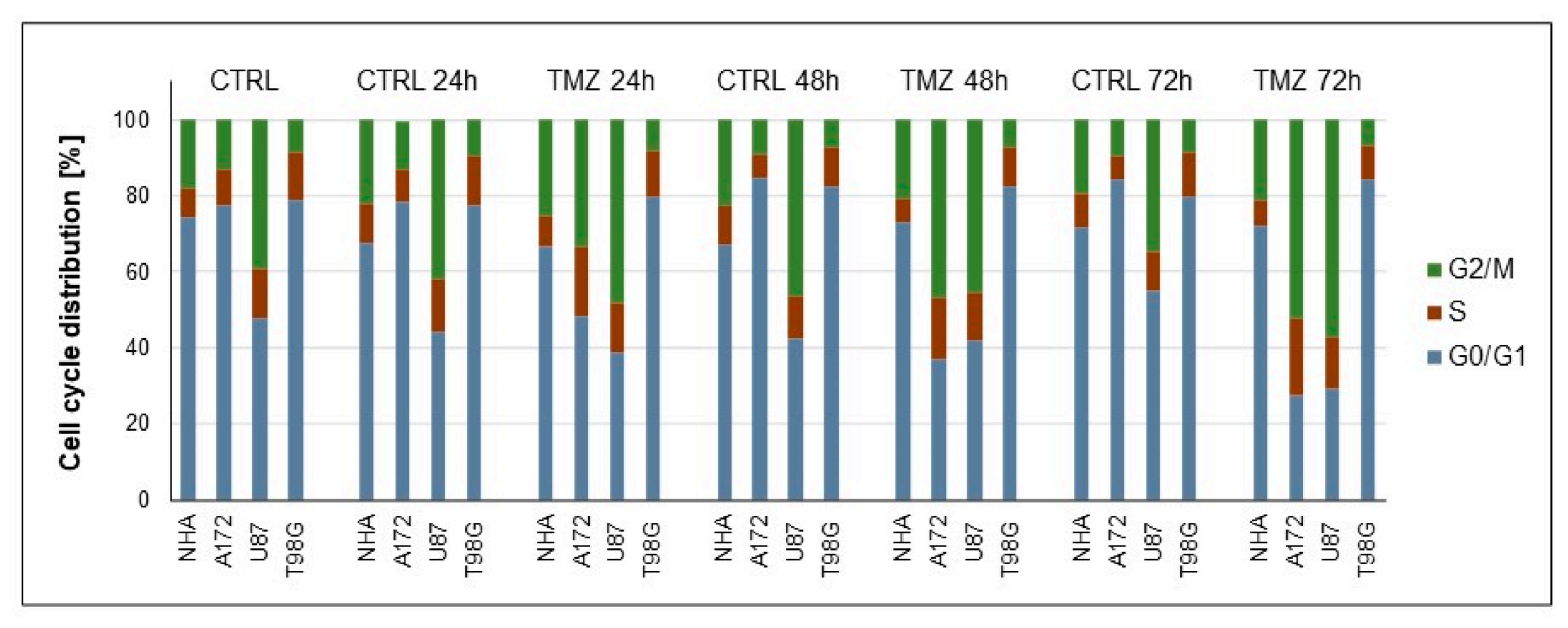
Cell cycle distribution in cell lines under control conditions and following TMZ treatment (350 µmol/L) at different times, showing proportions of cells in G0/G1 (blue), S (red), and G2/M (green) phases. Bars represent the mean values of three independent biological replicates.

Cell cycle distribution was analysed in normal NHA and GBM cell lines (A172, U87, T98G) following TMZ treatment for 24, 48, and 72 h. Gates were defined based on control samples for each biological replicate and kept constant across all time points and treatments to ensure accurate comparison. TMZ treatment induced only minor changes in cell cycle distribution in NHA cells. The G2/M population increased slightly at 24–72 h, accompanied by a decrease in G0/G1 phase, while the overall distribution remained relatively stable. These findings indicate limited sensitivity of non-tumour cells to TMZ and preservation of normal cell cycle regulation (Figure 2).

In A172 cells, TMZ treatment induced a pronounced and time-dependent accumulation in the G2/M phase. After 48–72 h, up to 45–55% of cells were arrested in G2/M, accompanied by a marked decrease in the G0/G1 population, indicating activation of DNA damage-induced cell cycle checkpoint (Figure 2). U87 cells exhibited a highly proliferative baseline profile, with a substantial proportion of cells already present in the G2/M phase. TMZ treatment induced a pronounced and time-dependent accumulation of U87 GBM cells in the G2/M phase. After 72 h, up to 55–60% of cells were arrested in G2/M, accompanied by a reduction in the G0/G1 population, suggesting an activation of DNA dam-age-induced cell cycle checkpoint, similar to A172 (Figure 2).

By contrast, T98G cells displayed minimal alterations in cell cycle distribution, suggesting an impaired or attenuated checkpoint response associated with resistance. No significant accumulation in the G2/M phase was observed, indicating a resistant phenotype (Figure 2). Unlike the T98G cells, whose cell cycle distribution changed minimally, both U87 and A172 cells showed strong G2/M arrest following TMZ treatment. TMZ induces a cell cycle arrest in sensitive GBM cells, whereas resistant cells fail to activate this response.

**Figure 3:**
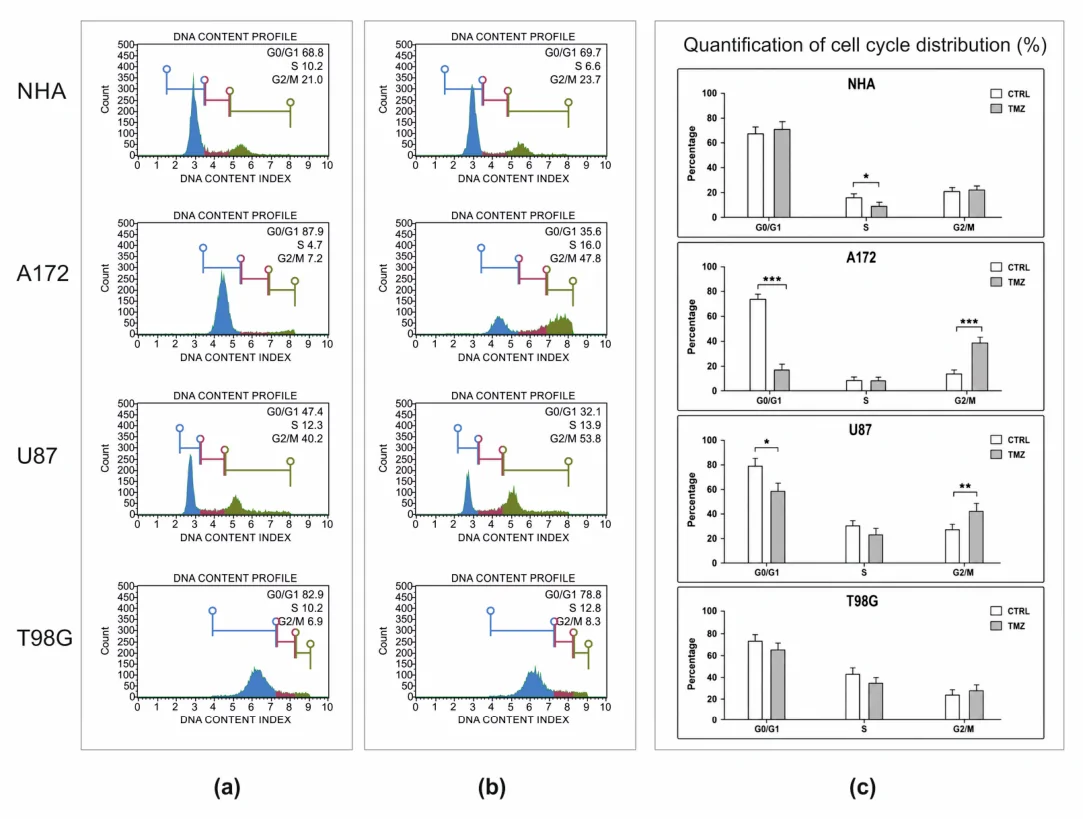
Representative DNA content histograms illustrating cell cycle distribution in all cell lines after 48 h incubation. Part (**a**) represents cell cycle phases under normal conditions (control cells without TMZ treatment); (**b**) represents cells treated with 350 µmol/L TMZ; (**c**) is quantitative interpretation of histograms. Individual phases of the cell cycle in histograms of column (**a**,**b**) are coloured as blue (G0/G1), red (S), and green (G2/M). Data in column (**c**) represent mean ± SD from three independent biological replicates. Statistical significance was determined using Welch’s two-sample *t*-test. CTRL—control group; TMZ—cells treated with temozolomide 350 μM; * *p* < 0.05, ** *p* < 0.01 and *** *p* < 0.001.

Overall, TMZ treatment results in a redistribution of cells across the cell cycle phases compared with untreated controls (Figure 3a,b). These observations support the concept that TMZ-sensitive GBM cells activate DNA damage responses more efficiently than resistant phenotypes (Figure 3c).

### 2.3. Protein MGMT Expression

**Figure 4:**
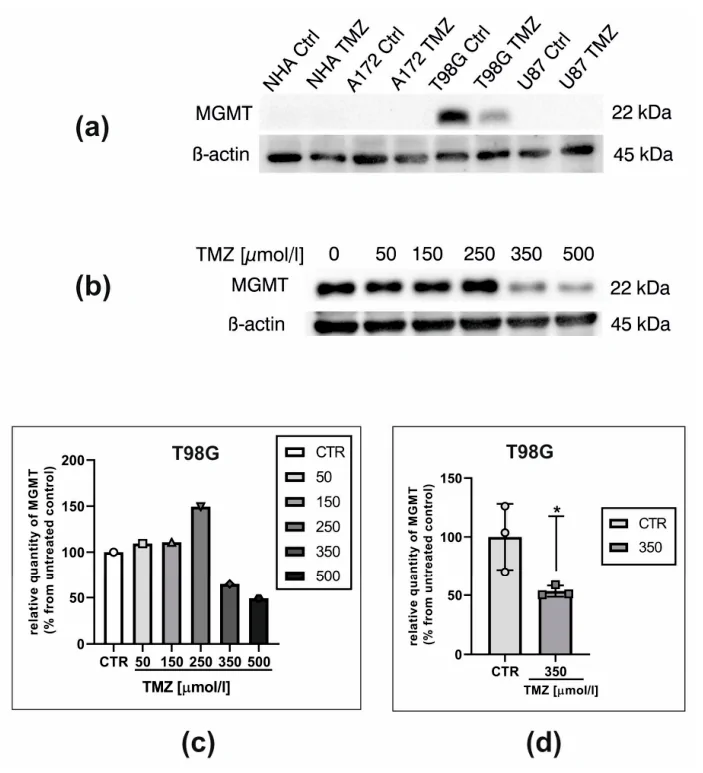
Temozolomide modulates MGMT protein expression in GBM cell lines. (**a**) Representative Western blot analysis of MGMT protein expression in NHA and GBM cell lines under control (Ctrl) conditions and following TMZ treatment. (**b**) Western blot showing MGMT expression in T98G cells treated with increasing concentrations of TMZ (0–500 μmol/L). (**c**) Densitometric quantification of MGMT protein levels in T98G cells treated with TMZ, expressed as a percentage relative to untreated control. Data indicate an increase in MGMT expression at intermediate concentrations (up to 250 μmol/L), followed by a marked decrease at higher concentrations. (**d**) Statistical analysis of MGMT expression in T98G cells comparing control and 350 μmol/L TMZ treatment. Data are presented as mean ± SD; * *p* < 0.05 versus control.

Protein expression of MGMT was analysed across four cell lines, similarly as in the previous section, following treatment with TMZ. Western blot analysis (Figure 4a) revealed minimal or undetectable MGMT expression in NHA and A172 cells under both control and TMZ-treated conditions. By contrast, T98G cells exhibited strong basal MGMT expression, which appeared reduced following TMZ treatment. U87 cells showed low MGMT expression with no substantial change upon treatment. β-actin was used as a loading control and for normalisation of intensities of observed bands. T98G cells were treated with increasing concentrations of TMZ (0–500 μmol/L) to further investigate the effect of TMZ on MGMT expression. As shown in Figure 4b, MGMT protein levels remained relatively stable at lower concentrations (50–250 μmol/L), with a slight increase observed at 250 μmol/L. However, a marked reduction in MGMT expression was evident at higher TMZ concentrations (350 and 500 μmol/L).

Overall, these results indicate that MGMT expression is cell line-dependent and that high concentrations of TMZ significantly downregulate MGMT protein levels in T98G cells.

### 2.4. LncRNA Expression Profiles

**Figure 5:**
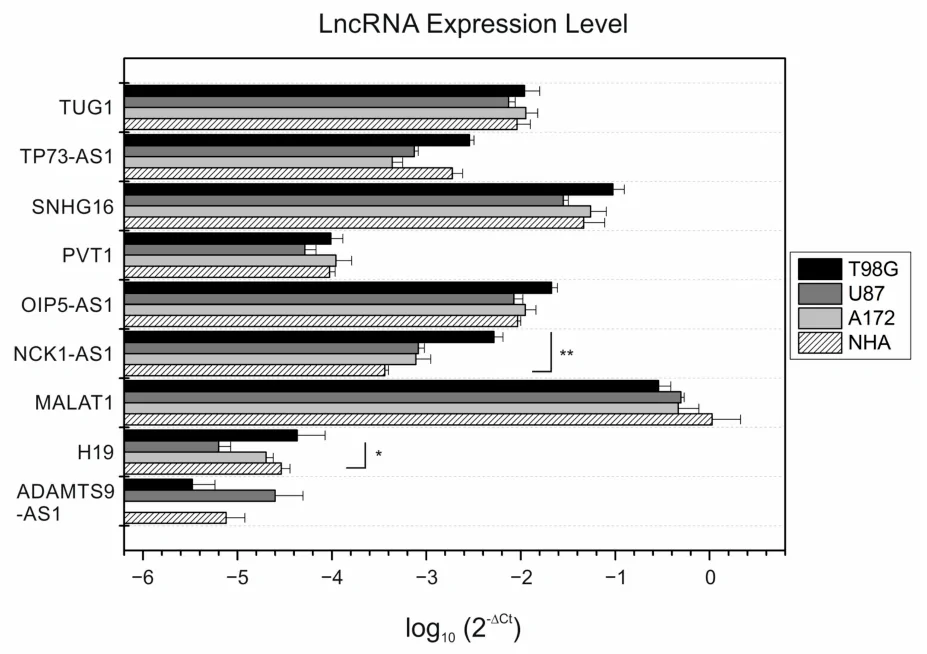
Absolute expression levels of selected lncRNAs in all cell lines. Expression is presented as log10-transformed values of relative quantification. Bars represent mean values, with error bars indicating standard deviation (±SD). Welch’s two sample non-parametric test was used to compare ΔCt dates of control and GBM cell lines. A minimum of three biological replicates were used for each gene of an individual cell line. Statistical significance between selected groups is indicated as * *p* < 0.05 and ** *p* < 0.01.

On the basis of publicly available data, we selected nine lncRNAs that play significant oncogenic or tumour-suppressor roles in various tumours: ADAMTS9-AS1, H19, MALAT1, NCK1-AS1, OIP5-AS1, PVT1, SNHG16, TP73-AS1, and TUG1 (Table 1). The expression and regulation patterns were analysed across three GBM cell lines (A172, U87, and T98G) and compared with NHAs. Both relative gene regulation (fold change) and absolute expression levels (log10[2^−ΔCt^]) were evaluated. Analysis of fold changes revealed substantial alterations in lncRNA regulation between GBM cell lines and astrocytoma controls. Several transcripts exhibited consistent downregulation, most notably MALAT1, which showed a marked decrease across all three GBM cell lines, with the strongest reduction observed in T98G. Similarly, H19 was downregulated in A172 and U87, although T98G was slightly upregulated. By contrast, NCK1-AS1 was significantly upregulated in all GBM cell lines, particularly in T98G, indicating a strong increase in its transcriptional activity (Figure 5).

Other lncRNAs demonstrated cell line-specific variability. ADAMTS9-AS1 was strongly upregulated in U87 but slightly downregulated in T98G, while no data were available for A172. OIP5-AS1 and PVT1 showed downregulation in A172 and U87, with a shift towards upregulation in T98G. Moderate upregulation was observed for SNHG16 across all cell lines. Conversely, TP73-AS1 was downregulated in A172 and U87 but upregulated in T98G, whereas TUG1 displayed mild upregulation in A172 and U87 followed by slight downregulation in T98G (Figure 5).

Evaluation of absolute expression levels supported these findings. Overall, most lncRNAs exhibited lower absolute expression (more negative values) in GBM cell lines compared with NHA controls. MALAT1 showed the highest expression among the analysed transcripts in all samples, although its levels remained slightly reduced in tumour cell lines relative to NHA. NCK1-AS1 and OIP5-AS1 displayed moderate expression with a tendency towards reduced levels in GBM cells (Figure 5). More pronounced decreases in absolute expression were observed for H19, ADAMTS9-AS1, and PVT1, particularly in T98G and U87. TP73-AS1 and TUG1 also showed reduced expression across GBM cell lines, although with some variability between individual lines. By contrast, SNHG16 exhibited relatively stable expression across all samples, with only minor differences between tumour and control cells (Figure 5). Statistical analysis of the expression data identified significant differences for H19 (*p* < 0.05) and NCK1-AS1 (*p* < 0.01), as indicated in Figure 5. Error bars reflect variability across replicates, demonstrating the consistency of the measurements.

Table 1 summarises the mean fold changes in lncRNA gene regulation in three GBM cell lines relative to normal astrocytoma cells. Both upregulation and downregulation patterns are observed across the analysed genes. Genes were considered significantly differentially regulated when the mean fold change was ≤−2.0 or ≥+2.0, with statistical significance defined by *p*-values < 0.05 (Welch’s two-sample *t*-test). Significant differences were observed for selected genes, as indicated by corresponding *p*-values.

Taken together, these results indicate that GBM cell lines are characterised by distinct lncRNA expression and regulatory profiles, with both consistent and cell line-specific alterations relative to normal astrocytes.

### 2.5. Effect of Temozolomide on LncRNA Expression

**Figure 6:**
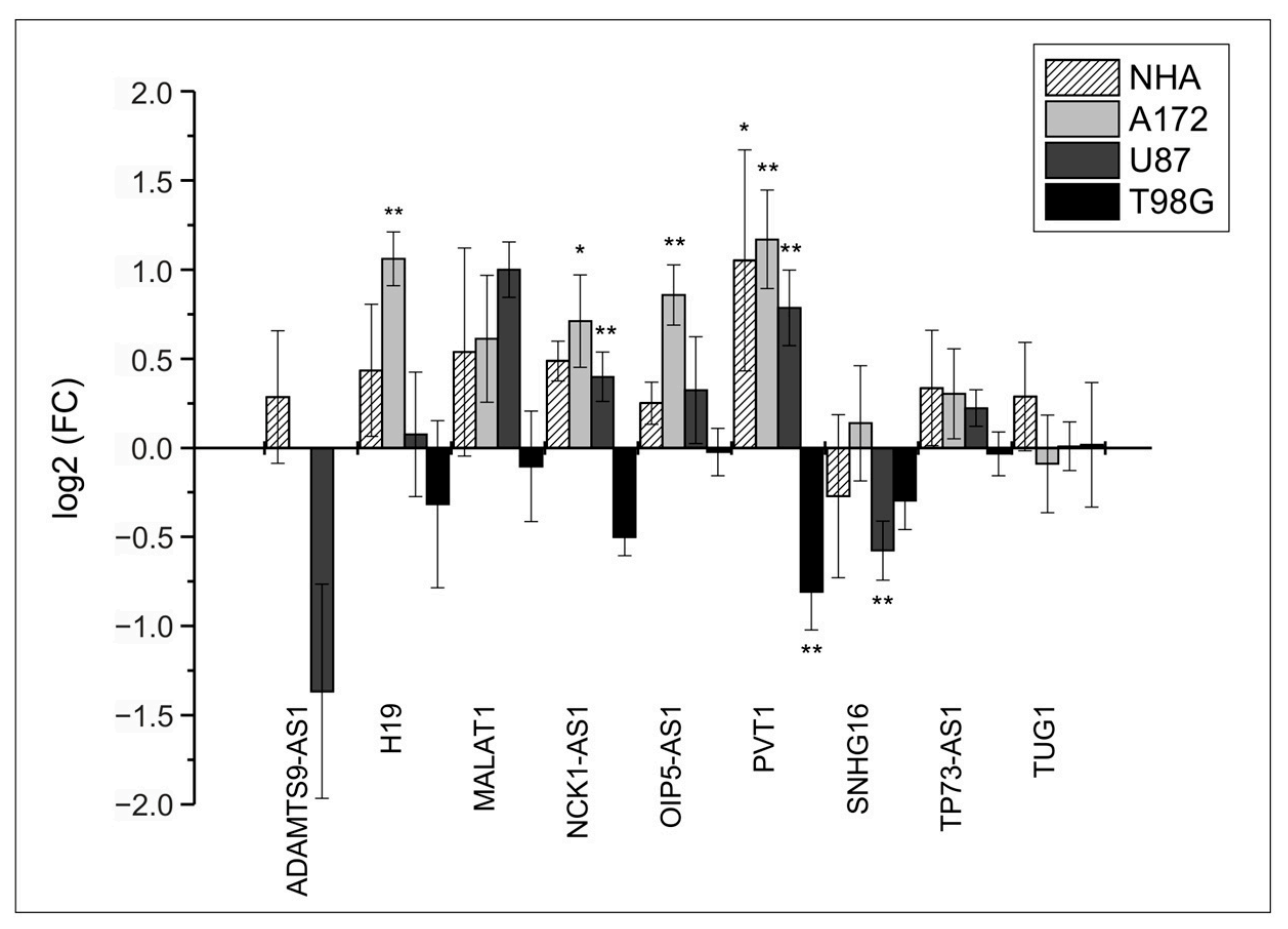
Comparison of the fold changes in selected lncRNA gene expression between the TMZ treated cell lines compared with untreated cells. Bar plots represent mean log_2_FC values, with error bars representing standard deviation (±SD). Positive values indicate upregulation, while negative values indicate downregulation relative to the reference. (* *p* < 0.05, ** *p* < 0.01).

The analysis of log2 fold changes (log_2_FC) revealed gene- and cell line-specific responses to TMZ treatment when compared with untreated controls. Overall, no uniform direction of regulation was observed across all lncRNAs, highlighting a heterogeneous transcriptional response to TMZ (Figure 6).

Among the analysed transcripts, PVT1 showed the most pronounced induction, particularly in A172 and NHA cells, with moderate upregulation also observed in U87, while T98G displayed decreased expression (Figure 6). This finding suggests a potential TMZ-responsive activation of PVT1 that may be context-dependent and attenuated in more resistant GBM phenotypes such as T98G. Similarly, H19 and OIP5-AS1 were significantly upregulated in A172 cells, with more moderate or variable changes in the remaining cell lines. The strong induction in A172 may indicate a higher transcriptional sensitivity to TMZ in this line (Figure 6). By contrast, SNHG16 was consistently downregulated across most GBM cell lines, with the most pronounced decrease in T98G and U87, suggesting a possible association between SNHG16 suppression and TMZ exposure. For NCK1-AS1, A172 and U87 increased, while T98G again exhibited downregulation, reinforcing the notion of a differential TMZ response depending on cellular background (Figure 6).

MALAT1 showed relatively stable or mildly increased expression across cell lines, indicating that its regulation may be less sensitive to TMZ or more tightly controlled. Notably, ADAMTS9-AS1 was strongly downregulated in U87 cells, whereas other lines showed minimal change, pointing to a cell line-specific effect. TP73-AS1 and TUG1 exhibited only modest changes, suggesting a limited response to TMZ under the tested conditions.

Importantly, T98G cells, often considered more TMZ-resistant, tended to show downregulation or weaker induction patterns across multiple lncRNAs (e.g., PVT1, NCK1-AS1, SNHG16), which may reflect an attenuated transcriptional response to treatment.

Taken together, these results demonstrate that TMZ induces distinct and gene-specific expression changes in lncRNAs, with clear variability between GBM cell lines. This heterogeneity may reflect differences in drug sensitivity, underlying molecular pathways, or resistance mechanisms.

## 3. Discussion

This study investigated the relationship between TMZ responsiveness and the expression of selected lncRNAs in GBM cell lines with distinct molecular and phenotypic characteristics. Our findings demonstrate marked heterogeneity in TMZ sensitivity among GBM models and reveal that several lncRNAs exhibit cell line-specific expression patterns associated with TMZ exposure and resistance mechanisms. These observations support the growing body of evidence suggesting that lncRNAs contribute to GBM progression and therapeutic adaptation [2,15,19,20].

We based our investigation on our previous molecular findings obtained in GBM cells [14,29], complemented by array-based profiling of cancer pathway mRNAs in a smaller cohort of patients with brain tumours [30]. Based on the preliminary results, we assume that lncRNA expression levels, both in distinct cell lines and in samples obtained from patients with different diagnoses, contribute to differential responses to TMZ therapy.

Consistent with previous studies, A172 cells displayed the greatest sensitivity to TMZ, whereas T98G cells exhibited a resistant phenotype characterised by sustained viability and limited cell cycle disruption following treatment [10,16,17]. The pronounced G2/M arrest observed in A172 and U87 cells following TMZ exposure is in agreement with the established mechanism of TMZ-induced DNA damage, which activates checkpoint signalling and disrupts mitotic progression [11,12].

The differential cell cycle responses observed among GBM cell lines further suggest that lncRNAs may contribute to TMZ sensitivity through modulation of DNA damage checkpoints and cell cycle regulation. The pronounced G2/M arrest detected in TMZ-sensitive cells is consistent with activation of DNA damage signalling, whereas resistant T98G cells-maintained cell cycle progression despite TMZ exposure, potentially due to efficient MGMT-mediated DNA repair [31].

MGMT expression patterns observed in this study further support its central role in TMZ resistance. T98G cells exhibited strong basal MGMT expression, while A172 and NHA cells showed minimal or undetectable levels. This finding is consistent with previous reports identifying MGMT activity as one of the principal determinants of GBM responsiveness to alkylating agents [11,16,17]. Several studies have demonstrated that MGMT expression levels strongly influence the sensitivity of GBM cells to TMZ treatment. High MGMT protein expression is generally associated with increased resistance to TMZ due to the enzyme’s ability to remove cytotoxic O6-methylguanine adducts generated by alkylating therapy [16,17]. By contrast, GBM cells with low MGMT expression or MGMT promoter methylation typically exhibit enhanced TMZ sensitivity and improved therapeutic response [8,11]. Previous studies demonstrated that T98G GBM cells exhibit high basal MGMT expression associated with intrinsic resistance to TMZ treatment. Zhang reported that T98G cells maintain strong MGMT protein expression and exhibit only limited sensitivity to TMZ exposure, particularly at lower concentrations, further supporting the role of MGMT in chemoresistance [32]. More recent findings by Li et al. further demonstrated that TMZ treatment induces progressive ubiquitination and degradation of MGMT in T98G cells, particularly at increasing TMZ concentrations and prolonged exposure times. These observations suggest that although MGMT initially contributes to TMZ resistance, higher TMZ doses may overcome this protective mechanism through depletion or destabilisation of the MGMT protein [33]. Our findings are in agreement with these observations, as the TMZ-resistant T98G cells displayed strong basal MGMT expression, whereas the more TMZ-sensitive A172 cells exhibited minimal MGMT protein levels. Furthermore, the reduced MGMT expression following exposure to higher TMZ concentrations may indicate treatment-induced depletion of MGMT-mediated DNA repair capacity or adaptive cellular stress responses. Although MGMT remains one of the principal determinants of TMZ responsiveness, accumulating evidence indicates that MGMT-independent resistance mechanisms also substantially contribute to GBM treatment failure. These mechanisms include dysregulation of mismatch repair pathways, enhanced base excision repair, altered apoptosis signalling, autophagy activation, metabolic reprogramming, and epigenetic modifications mediated by non-coding RNAs. Although several lncRNAs displayed expression patterns consistent with MGMT-associated TMZ resistance, the present study was not designed to establish direct regulatory relationships. Therefore, these observations should be interpreted as associations that require validation using functional gain- and loss-of-function experiments. Several lncRNAs have recently been shown to regulate these pathways simultaneously, highlighting their role as integrative modulators of chemoresistance rather than isolated transcriptional regulators [23]. One limitation of the present study is that MGMT promoter methylation was not experimentally determined. Although the MGMT promoter methylation status of the widely used glioblastoma cell lines A172, U87 and T98G has been reported previously in several independent studies [34,35,36], the primary objective of the present work was to investigate the relationship between MGMT protein expression, TMZ responsiveness and lncRNA expression profiles rather than the epigenetic regulation of MGMT. Future studies integrating MGMT promoter methylation, MGMT protein expression, and lncRNA profiling would provide a more comprehensive understanding of the molecular mechanisms underlying temozolomide resistance. In addition, recent studies have demonstrated that temozolomide induces time- and dose-dependent ubiquitination and proteasomal degradation of MGMT, thereby contributing to depletion of DNA repair capacity and modulation of cellular sensitivity to alkylating therapy [37,38]. However, investigation of MGMT ubiquitination and proteasome-mediated degradation requires dedicated biochemical approaches, including ubiquitination assays, immunoprecipitation, and proteasome inhibition experiments, which were beyond the scope of the present study.

The observed heterogeneity between GBM cell lines further reflects the highly plastic and adaptive nature of GBM biology. Recent evidence suggests that GBM cells dynamically switch between proliferative and therapy-resistant states in response to chemotherapy-induced stress, with lncRNAs acting as critical regulators of these transitions. In particular, lncRNAs have been implicated in the maintenance of GBM stem cell phenotypes, epithelial-to-mesenchymal transition-like programmes, metabolic adaptation, and modulation of DNA damage response pathways, all of which contribute to therapeutic failure and tumour recurrence [39]. Among the analysed lncRNAs, several transcripts demonstrated notable dysregulation in GBM cells compared with normal astrocytes. MALAT1 expression was consistently reduced in all GBM cell lines relative to NHA controls, despite previous reports frequently describing MALAT1 as an oncogenic lncRNA associated with glioma progression and chemoresistance [13]. Although MALAT1 expression was reduced in our GBM models relative to normal astrocytes, previous studies demonstrated that TMZ exposure itself may induce MALAT1 expression through NF-κB- and p53-dependent signalling pathways. Voce et al. demonstrated that TMZ treatment increases MALAT1 transcription and promotes mesenchymal transformation associated with chemoresistance in GBM cells [18]. These findings suggest that MALAT1 regulation may depend not only on intrinsic tumour subtype differences, but also on treatment duration, stress adaptation, and the acquisition of resistant cellular states. LncRNA plasmacytoma variant translocation 1 (PVT1), H19, and OIP5-AS1 demonstrated highly variable regulation depending on the cellular background. The strong induction of PVT1 and H19 in A172 cells following TMZ treatment may indicate that compensatory survival pathways in TMZ-sensitive cells are activated [22,23,26]. Although only one TMZ exposure condition (350 µmol/L, 48 h) was used for the molecular analyses, this condition was selected following comprehensive dose–response experiments performed at three exposure times (24, 48, and 72 h). Future studies should investigate the temporal dynamics of lncRNA regulation during both early and prolonged TMZ exposure. From a broader perspective, the results reinforce the idea that lncRNAs participate in multiple regulatory networks that influence GBM progression and chemoresistance [2,27,28].

Our findings are consistent with recent studies demonstrating the important contribution of lncRNAs to TMZ resistance in GBM. In particular, H19, MALAT1, and PVT1 have repeatedly been identified as regulators of glioma progression and adaptive responses to TMZ treatment [2]. Chen et al. recently showed that lncRNA H19 is significantly upregulated in TMZ-resistant GBM cells and tissues, while silencing of H19 restored TMZ sensitivity and enhanced apoptosis in resistant cells. The authors further demonstrated that H19 mediates TMZ resistance through the miR-138-5p/miR-22-3p/BMP2 regulatory axis, suggesting its direct involvement in chemoresistance-associated signalling pathways [11]. Importantly, H19 silencing restored TMZ sensitivity both in vitro and in vivo, supporting its potential therapeutic relevance in resistant GBM.

The present findings suggest that different lncRNAs may contribute to distinct aspects of TMZ responsiveness. Whereas H19, PVT1 and OIP5-AS1 have previously been implicated in adaptive responses to temozolomide treatment [2,40,41], NCK1-AS1 was predominantly associated with the intrinsically resistant T98G phenotype in our experimental model, suggesting a potential role in innate resistance. These observations indicate that individual lncRNAs may differentially participate in intrinsic and therapy-induced resistance mechanisms, although their precise functional interactions require further mechanistic investigation.

Moreover, several recent reviews highlighted that lncRNAs regulate multiple biological processes associated with TMZ resistance, including epithelial–mesenchymal transition, DNA damage repair, apoptosis evasion, glioma stem cell maintenance, and modulation of MGMT-related pathways. These observations support our findings demonstrating heterogeneous lncRNA responses among GBM cell lines with different TMZ sensitivities [2,33]. Importantly, the variability observed between A172, U87, and T98G cells in this study further supports the concept that lncRNA-mediated regulation of TMZ resistance is highly context-dependent and influenced by the molecular background of individual GBM subtypes. From a translational perspective, lncRNAs are attractive candidates for future precision oncology strategies in GBM. With their tissue-specific expression profiles, relative stability, and detectability in liquid biopsies, several lncRNAs have been proposed as prognostic biomarkers or predictors of TMZ responsiveness. Moreover, therapeutic approaches targeting oncogenic lncRNAs using antisense oligonucleotides, siRNA-based systems, or CRISPR-associated technologies are currently under intensive investigation [42].

Although lncRNAs represent attractive therapeutic targets, several important challenges remain before their clinical application. These include efficient delivery across the blood–brain barrier, potential off-target effects, tumour heterogeneity and long-term safety of antisense oligonucleotide or siRNA-based therapies. Therefore, additional preclinical studies are required before lncRNA-targeted interventions can be translated into clinical practice. We would also like to point out the limitations of the study. First, all experiments were performed in established GBM cell lines, which only partially reproduce the complexity and heterogeneity of human glioblastoma. Second, MGMT promoter methylation and post-translational MGMT regulation were not investigated. Third, apoptosis-specific assays were not included, and therefore the precise mechanisms of TMZ-induced cytotoxicity remain to be elucidated. Finally, the findings require validation in patient-derived glioma models, orthotopic animal models and clinical specimens before their translational significance can be fully established.

Future studies should validate these findings in additional glioblastoma models, including established cell lines, patient-derived glioblastoma stem cells, three-dimensional organoid systems, and clinical tumour specimens. Such studies will be essential to determine the biological significance, translational relevance, and potential clinical utility of the identified lncRNAs as predictive biomarkers and therapeutic targets for temozolomide resistance.

## 4. Materials and Methods

### 4.1. Cell Culture and Treatment

The glioma tumour cell panel (T98G, A172 and U87) were purchased from the American Type Culture Collection (ATCC, Manassas, VA, USA) under catalogue numbers: CRL-1690™, CRL-1620™, HTB-14™; respectively. The biological characteristics and molecular background of the glioblastoma cell lines employed in this study are summarized in Table 2, providing the rationale for their selection as representative models of different temozolomide response phenotypes. The normal human astrocytes (NHA) were purchased from Lonza Group (CC-2565; Basel, Switzerland) and cultured in Dulbecco’s Modified Eagle Medium/Nutrient Mixture F-12 (DMEM/F-12, 1:1; Gibco, Thermo Fisher Scientific, Paisley, UK) supplemented with 10% fetal bovine serum (FBS-HI-12A, Capricorn Scientific, Ebsdorfergrund, Germany) and 1% (*v*/*v*) penicillin/streptomycin solution (Pen/Strep, PS-B 100x, Capricorn Scientific, Ebsdorfergrund, Germany).

The human glioblastoma cell lines were cultured in High-Glucose DMEM (Capricorn Scientific, Ebsdorfergrund, Germany) supplemented with 10% FBS-HI and 1% Pen/Strep. All cell lines were cultured at 37 °C in a humidified incubator containing 5% CO_2_, and the culture medium was replaced every three days. Prior to each experiment, adherent cells were detached using 0.25% trypsin–EDTA solution containing phenol red (T4049; Sigma-Aldrich, St. Louis, MO, USA) to obtain a single-cell suspension. Cell numbers were determined using a Countess™ automated cell counter (Invitrogen, Carlsbad, CA, USA).

Unless otherwise stated, all experiments were performed using three independent biological replicates. Cell culture experiments were repeated independently on different days using freshly prepared cell cultures. Technical replicates were included where appropriate for each analytical method and are specified in the corresponding Materials and Methods subsections. Data are presented as mean ± standard deviation (SD) or standard error of the mean (SEM), as indicated in the figure legends.

Temozolomide (TMZ; HY-17364; MedChemExpress, Monmouth Junction, NJ, USA) was dissolved in dimethyl sulfoxide (DMSO) to prepare a 50 mmol/L stock solution. Cells were treated with final TMZ concentrations of 250 µmol/L or 350 µmol/L in 25 cm^2^ culture flasks for 48 h. Following treatment, the confluency of TMZ-treated cells ranged between 60 and 75%, whereas untreated control cells reached approximately 90% confluency.

### 4.2. Cell Viability Assay

Cell viability was evaluated using the colorimetric 3-(4,5-dimethylthiazol-2-yl)-2,5-diphenyltetrazolium bromide (MTT) assay, which is based on the enzymatic reduction in MTT to purple formazan crystals. NHA cells were seeded into 96-well plates at a density of 5.0 × 10^3^ cells/well, whereas glioblastoma cell lines were seeded at 3.0 × 10^3^ cells/well. After 24 h, the culture medium was replaced with fresh medium containing increasing concentrations of TMZ, followed by incubation for an additional 24–72 h.

At each experimental time point (24, 48, and 72 h), the medium was aspirated and replaced with 100 µL of fresh culture medium containing MTT solution (0.5 mg/mL). Cells were incubated for 5 h under standard culture conditions. Subsequently, 100 µL of 5% (*w*/*v*) SDS solution was added to dissolve the generated formazan crystals during overnight incubation (12–16 h). Absorbance was measured at 540 nm using a Synergy™ H4 microplate reader (BioTek Instruments, Agilent Technologies, Winooski, VT, USA). Cell viability was expressed as a percentage relative to untreated controls, and IC_50_ values were calculated. Each biological replicate consisted of six technical replicates for each TMZ concentration and time point. The complete experiment was independently repeated three times. The concentration of 350 µmol/L TMZ for 48 h was selected for subsequent molecular analyses based on the results of the preliminary dose–response experiments, which demonstrated reproducible cytotoxic effects while preserving sufficient viable cells for protein and RNA analyses.

### 4.3. Cell Cycle Analysis

Cell cycle distribution was analysed using the Muse™ Cell Cycle Assay Kit in combination with the Muse™ Cell Analyzer (Luminex Corporation, Austin, TX, USA) according to the manufacturer’s instructions. Following exposure to 350 µmol/L TMZ for 0, 24, 48, and 72 h, cells were detached using 0.25% trypsin–EDTA solution containing phenol red (T4049; Sigma-Aldrich, St. Louis, MO, USA) for 3 min at 37 °C. Cells were subsequently washed with DPBS and centrifuged at 300× *g* for 5 min at 13 °C. Cell pellets were rinsed with DPBS and fixed in cold 70% (*v*/*v*) ethanol. Fixed cells were stored at −20 °C until analysis. Prior to analysis, fixed cells were incubated with Muse™ Cell Cycle reagent for 30 min at room temperature in the dark. The proportions of cells in the G0/G1, S, and G2/M phases were quantified. The proliferation index (PI) was calculated according to the following equation:

PI (%) = [(S + G2/M)/(G0/G1 + S + G2/M)] × 100.

### 4.4. Western Blot Analysis

Protein lysates were prepared from cells cultured and treated as described above. Following three washes with DPBS, proteins were extracted using RIPA lysis buffer (89900; Thermo Fisher Scientific, Paisley, UK) supplemented with Halt™ protease inhibitor cocktail (78430; Thermo Fisher Scientific, Paisley, UK), sodium orthovanadate (1.0 µmol/L), and sodium fluoride (50.0 mmol/L). Cell lysis was performed according to the manufacturer’s instructions. Protein concentration was determined using the DC Protein Assay (5000111; Bio-Rad Laboratories, Hercules, CA, USA) with pre-diluted bovine serum albumin (BSA) standards (23208; Thermo Fisher Scientific, Paisley, UK). Equal amounts of protein (30 µg per sample) were separated on 12% SDS–PAGE gels and subsequently transferred onto nitrocellulose membranes using the Trans-Blot^®^ Turbo™ Transfer System (Bio-Rad Laboratories, Hercules, CA, USA).

Membranes were incubated with primary antibodies against MGMT (1:1000; MA5.13506; Thermo Fisher Scientific, Paisley, UK) and β-actin (1:1000; #3700; Cell Signaling Technology, Danvers, MA, USA). After incubation with the appropriate secondary antibodies—anti-mouse (1:10,000; A0168; Sigma-Aldrich, St. Louis, MO, USA) or anti-rabbit (1:30,000; A9169; Sigma-Aldrich, St. Louis, MO, USA)—protein bands were visualised using SuperSignal™ West Pico chemiluminescent substrate (Thermo Fisher Scientific, Paisley, UK) and detected using the ChemiDoc™ XRS imaging system (Bio-Rad Laboratories, Hercules, CA, USA). Band intensities were quantified using Quantity One software (Bio-Rad Laboratories, Hercules, CA, USA) and normalised to β-actin expression.

### 4.5. RNA Extraction and cDNA Synthesis

Total RNA was isolated from frozen cell pellets (0.5–2.0 × 10^6^ cells) using the RNeasy Mini Kit (74104; QIAGEN GmbH, Hilden, Germany) according to the manufacturer’s protocol. RNA purity and concentration were assessed using a P300 NanoPhotometer^®^ (Implen GmbH, Munich, Germany). Subsequently, 2 µg of total RNA was reverse-transcribed into single-stranded cDNA using the RT^2^ First Strand Kit (330401; QIAGEN GmbH, Hilden, Germany following the manufacturer’s instructions.

### 4.6. Quantitative Real-Time PCR and Data Analysis

Quantitative real-time PCR (qRT-PCR) was performed using TB Green^®^ Premix Ex Taq™ II (RR82LR; Takara Bio Inc., Kusatsu, Shiga, Japan). PCR reaction mixtures were prepared according to the manufacturer’s recommendations and dispensed into 96-well plates at a final volume of 20 µL per well. Plates were briefly centrifuged at 1000× *g* for 1 min prior to amplification. Amplification was carried out using the ViiA™ 7 Real-Time PCR System (Applied Biosystems, Foster City, CA, USA). Following an initial denaturation step at 95 °C for 10 min, amplification was performed over 40 cycles consisting of denaturation at 95 °C for 15 s and annealing/extension at 60 °C for 1 min. Gene expression analysis was performed using cDNA obtained from three independent biological replicates. Each biological replicate was analysed in duplicate PCR reactions, and mean Ct values were used for subsequent statistical analysis. Expression levels of target genes were normalised to two endogenous reference genes, ACTB (β-actin) and B2M (β-2-microglobulin). Primer sequences are summarised in Table S1. Absolute expression levels of investigated lncRNAs were calculated using the ΔCt method relative to housekeeping genes and expressed as (log10[2^−ΔCt^]) values. Relative gene expression was determined using the ΔΔCt method and expressed as log_2_ fold-change (FC) values. Genes with an average fold change ≤−2.0 or ≥2.0 and a corresponding *p*-value < 0.05 were considered differentially expressed.

### 4.7. Statistical Analysis

Statistical analyses of gene expression data were performed using OriginPro 8.5 software (OriginLab Corporation, Northampton, MA, USA). Welch’s two-sample non-parametric test was used to compare ΔCt values between control and glioblastoma cell lines. Differences between treated and untreated groups within each cell line were evaluated using the two-sided Mann–Whitney U test. Statistical analyses were performed using log2-transformed relative quantification values. A *p*-value < 0.05 was considered statistically significant.

MGMT protein levels in T98G cells were quantified by densitometric analysis using Quantity One software (Bio-Rad Laboratories, Hercules, CA, USA) and normalised to the corresponding β-actin levels. Differences between treated and untreated samples were assessed using the unpaired Student’s T-test. Data were obtained from three independent experiments, and statistical significance was defined as *p* < 0.05.

## 5. Conclusions

This study demonstrates that GBM cell lines exhibit marked heterogeneity in their response to TMZ treatment, as reflected by differences in cell viability, cell cycle regulation, MGMT expression, and lncRNA transcriptional profiles. TMZ-sensitive cell lines, particularly A172, displayed pronounced G2/M arrest and greater cytotoxic responses, whereas the resistant T98G cells maintained high basal MGMT expression and showed limited alterations following treatment. Among the analysed lncRNAs, H19, MALAT1, NCK1-AS1, and PVT1 emerged as the transcripts most strongly associated with TMZ exposure and resistance-related phenotypes. Future research should integrate comprehensive epigenetic profiling, functional lncRNA knockdown or overexpression studies, patient-derived glioblastoma stem cell models, and liquid biopsy-based validation to determine whether the identified lncRNAs may serve as promising candidate biomarkers requiring validation in clinical specimens for precision treatment of glioblastoma.

Collectively, these findings support the growing evidence indicating that lncRNAs participate in key regulatory networks involved in GBM progression and chemoresistance. Selected lncRNAs may therefore represent promising biomarkers of TMZ responsiveness and potential molecular targets for future therapeutic strategies. Further studies that use patient-derived GBM models and clinical samples will be essential to validate their translational relevance.

## Figures and Tables

**Table 1:** Fold changes in lncRNA expression in glioblastoma cell lines relative to normal human astrocytes.

Symbol of Gene	Cell Line			Gene Description
	A172	T98G	U87	
*ADAMTS9-AS1*	N/A	−1.46	5.31 *	A disintegrin and metalloproteinase with thrombospondin type 1 motif 9
*H19*	−1.71	1.08	−4.78 **	Imprinted maternally expressed transcript
*MALAT1*	**−2.77**	**−8.24**	**−4.12**	Metastasis-associated lung adenocarcinoma transcript 1
*NCK1-AS1*	**2.30**	18.71 ***	2.32 ***	Non-catalytic region of tyrosine kinase adaptor protein 1
*OIP5-AS1*	−1.33	2.39 ***	−1.31	Opa interacting protein 5 antisense-1
*PVT1*	−1.14	1.68	−2.08 **	Plasmacytoma variant translocation 1
*SNHG16*	**2.59**	3.48 *	1.14	Small nucleolar RNA host gene 16
*TP73-AS1*	−3.16 **	1.60 *	−1.76 *	Tumor protein P73 antisense 1
*TUG1*	**2.23**	−1.08	1.46	Taurine upregulated gene 1

Data represent mean fold-change values calculated relative to NHA cells. Statistical significance was evaluated using Welch’s two-sample *t*-test (* *p*  <  0.05, ** *p*  <  0.01, *** *p*  <  0.001; N/A—not applicable). Fold-change values and statistical significance were evaluated independently; therefore, not all fold changes exceeding ≤−2.0 or ≥+2.0 (marked in bold) reached statistical significance.

**Table 2:** Biological characteristics of the human glioblastoma cell lines used in this study. The selected models represent different molecular backgrounds and distinct levels of intrinsic sensitivity to temozolomide (TMZ).

Cell Line	Molecular Characteristics *	MGMT Expression	TMZ Sensitivity	Rationale for Inclusion in This Study
A172	TP53 mutant, PTEN wild-type, relatively less invasive, rapid proliferation	Low/ undetectable	High (TMZ-sensitive)	Representative model of TMZ-sensitive glioblastoma with limited MGMT-mediated resistance.
U87	PTEN deficient, TP53 wild-type, moderate proliferation and invasiveness	Low/ undetectable	Moderate	Widely used reference glioblastoma model representing an intermediate response to TMZ and commonly employed in experimental neuro-oncology.
T98G	TP53 mutant, highly invasive, slower proliferation, DNA damage tolerant	High	Low (TMZ-resistant)	Representative model of intrinsic TMZ resistance characterized by constitutively high MGMT expression.

* Selected biological characteristics relevant to the present study.

## Data Availability

The original contributions presented in this study are included in the article/Supplementary Material. Further inquiries can be directed to the corresponding author.

## Supplementary Materials

The following supporting information can be downloaded at https://www.mdpi.com/article/10.3390/ph19081159/s1: Table S1: List of oligonucleotides used in this study.

## Abbreviations

The following abbreviations are used in this manuscript:

ACTB	β-actin
ADAMTS9-AS1	A disintegrin and metalloproteinase with thrombospondin type 1 motif 9
B2M	Beta-2 microglobulin
EDTA	Ethylenediaminetetraacetic acid
EGFR	Epidermal Growth Factor Receptor
GBM	Glioblastoma
H19	Imprinted maternally expressed transcript
IDH	Isocitrate dehydrogenase
lncRNAs	Long non-coding RNAs
MALAT1	Metastasis-associated lung adenocarcinoma transcript 1
MGMT	O6-methylguanine-DNA methyltransferase
MTT	3-[4,5-dimethylthiazol-2-yl]-2,5 diphenyl tetrazolium bromide
NCK1-AS1	Non-catalytic region of tyrosine kinase adaptor protein 1
NHA	Normal Human Astrocytes
OIP5-AS1	Opa interacting protein 5 antisense-1
PTEN	Platelet-Derived Growth Factor
PVT1	Plasmacytoma variant translocation 1
SDS	Sodium dodecyl sulfate
SNHG16	Small nucleolar RNA host gene 16
TP73-AS1	Tumor protein P73 antisense 1
TUG1	Taurine upregulated gene 1
TMZ	Temozolomide
WHO	World Health Organization
