# Supplementary material for: Association Between Temozolomide Resistance and Long Non-Coding RNA Expression Profiles in Glioblastoma Cell Lines
Source: Pharmaceuticals (Basel). 2026 Jul 25;19(8):1159. doi: 10.3390/ph19081159 (PMC13517041; doi:10.3390/ph19081159)
Supplement: Supplementary file 1 [file pharmaceuticals-19-01159-s001.zip › pharmaceuticals-4384098-supplementary.pdf]

Table S1. List of oligonucleotides used in this study.

Name of primer	Oligonucleotide sequence (5′→ 3′)	Direction
ADAMTS9-AS1	TCTGTTGCCCATTTCCTACC	Forward
	CCCTTCCATCCTGTCTACTCTA	Reverse
H19	TTTCATCCTTCTGTCTCTTTGT	Forward
	CAACCAGTGCAAATGACTTAG	Reverse
MALAT1	GACTTCAGGTCTGTCTGTTCT	Forward
	CAACAATCACTACTCCAAGC	Reverse
NCK1-AS1	GCCGCAGGAGAGACTTAACA	Forward
	CCTTCGGCTGGGATGACATT	Reverse
OIP5-AS1	TGCGAAGATGGCGGAGTAAG	Forward
	TAGTTCCTCTCCTCTGGCCG	Reverse
PVT1	GCCCCTTCTATGGGAATCACTA	Forward
	GGGGCAGAGATGAAATCGTAAT	Reverse
SNHG16	CAGAATGCCATGGTTTCCCC	Forward
	TGGCAAGAGACTTCCTGAGG	Reverse
TP73-AS1	CCGGTTTTCCAGTTCTTGCAC	Forward
	GCCTCACAGGGAAACTTCATGC	Reverse
TUG1	TAGCAGTTCCCCAATCCTTG	Forward
	CACAAATTCCCATCATTCCC	Reverse
ACTB*	CACGGCATCGTCACCAACT	Forward
	AGCCACACGCAGCTCATTG	Reverse
B2M*	GCTCGCGCTACTCTCTCTTT	Forward
	TCTGAATGCTCCACTTTTTCAA	Reverse

*Housekeeping genes
